# Osteocytic Connexin Hemichannels Modulate Oxidative Bone Microenvironment and Breast Cancer Growth

**DOI:** 10.3390/cancers13246343

**Published:** 2021-12-17

**Authors:** Yi Tian, Manuel A. Riquelme, Chao Tu, Yumeng Quan, Xiaowen Liu, Lu-Zhe Sun, Jean X. Jiang

**Affiliations:** 1 Department of Biochemistry and Structural Biology, University of Texas Health Science Center, San Antonio, TX 78229, USA; tianyi4978@bjhmoh.cn (Y.T.); Riquelme@uthscsa.edu (M.A.R.); tuchao@csu.edu.cn (C.T.); quanyumeng11@stu.xjtu.edu.cn (Y.Q.); xiaowenlable@gmail.com (X.L.); 2 Department of Thoracic Surgery, The Second Xiangya Hospital, Central South University, Changsha 410011, China; 3 Department of Cell Systems and Anatomy, University of Texas Health Science Center, San Antonio, TX 78229, USA; SUNL@uthscsa.edu

**Keywords:** Cx43 hemichannels, breast cancer bone metastasis, oxidative stress, estrogen deficiency

## Abstract

**Simple Summary:**

Estrogen deficiency with increased oxidative stress is associated with various pathological conditions, including cancers. Oxidative stress (OS) is elevated in the bone microenvironment with estrogen deficiency, but the impact of OS on tumor growth in bone remains largely unknown. In our research, we found that elevated oxidative stress inhibits tumor cell growth and the lack of Cx43 hemichannel function in bone osteocytes could mediate this effect. Impairment of Cx43 hemichannels either under oxidative stress or in Cx43-hemichannel-impaired transgenic mice under estrogen deficiency led to the inhibition of breast cancer growth. Treatment with an antioxidant reverses the effect, showing an increase in tumor growth in mice. This study unveiled a new mechanism regarding the role of osteocytic Cx43 hemichannels in modulating an oxidative bone microenvironment and the response of breast cancer cells to local oxidative stress.

**Abstract:**

Osteocytes, the most abundant bone cell types embedded in the mineral matrix, express connexin 43 (Cx43) hemichannels that play important roles in bone remodeling and osteocyte survival. Estrogen deficiency decreases osteocytic Cx43 hemichannel activity and causes a loss in osteocytes’ resistance to oxidative stress (OS). In this study, we showed that OS reduced the growth of both human (MDA-MB-231) and murine (Py8119) breast cancer cells. However, co-culturing these cells with osteocytes reduced the inhibitory effect of OS on breast cancer cells, and this effect was ablated by the inhibition of Cx43 hemichannels. Py8119 cells were intratibially implanted in the bone marrow of ovariectomized (OVX) mice to determine the role of osteocytic Cx43 hemichannels in breast cancer bone metastasis in response to OS. Two transgenic mice overexpressing dominant-negative Cx43 mutants, R76W and Δ130-136, were adopted for this study; the former inhibits gap junctions while the latter inhibits gap junctions and hemichannels. Under normal conditions, Δ130-136 mice had significantly more tumor growth in bone than that in WT and R76W mice. OVX increased tumor growth in R76W but had no significant effect on WT mice. In contrast, OVX reduced tumor growth in Δ130-136 mice. To confirm the role of OS, WT and Δ130-136 mice were administered the antioxidant N-acetyl cysteine (NAC). NAC increased tumor burden and growth in Δ130-136 mice but not in WT mice. Together, the data suggest that osteocytes and Cx43 hemichannels play pivotal roles in modulating the oxidative microenvironment and breast cancer growth in the bone.

## 1. Introduction

Bone metastasis, caused by cancer progression, usually happens at advanced stages of breast cancer [1]. Bone metastases can be deadly and cause major complications that greatly affect patients’ quality of life [2,3]. Recently, the cancer microenvironment in distal metastasized organs and tissues has been considered as a major factor in tumor progression [4,5,6]. Osteocytes, which represent about 95% of all bone cells and maintain the bone microenvironment, play an important role in mediating bone mechanical sensitivity and maintaining homeostasis and remodeling by coordinating osteoclast and osteoblast activity [7,8]. Additionally, the complex intercellular communication between osteocytes and other bone cells plays a critical role in bone remodeling. For example, osteocytes release factors that facilitate bone remodeling through the coupling of bone formation by osteoblasts immediately following the bone resorption by osteoclasts [9]. Osteocytes richly express connexins to form gap junctions and hemichannels (halves of gap junctions). Unlike gap junctions, which mediate intercellular communication between adjacent cells, hemichannels facilitate the exchange of small molecules (<1.2 kDa) between the cell and the extracellular microenvironment [10]. Connexin 43 (Cx43), a gap junction/hemichannel-forming protein, is a major connexin subtype expressed in bone cells and plays a crucial role in bone function, including osteoblast proliferation and differentiation, osteocyte mechanotransduction and survival, as well as bone development and bone mass after birth [11,12].

We have previously reported that impaired osteocytic hemichannels in Cx43 transgenic mice increased the number of apoptotic osteocytes [13]. Moreover, osteocytic Cx43 hemichannels protect osteocytes against estrogen-deficiency-induced apoptosis and bone loss [14]. Additionally, Cx43 hemichannels protect cultured osteocytes against high oxidative stress (OS)–induced cell death [15]. Collectively, these results implicate an important role of Cx43 and specifically Cx43 hemichannels in osteocyte viability. Furthermore, we have shown that the opening of hemichannels and the release of ATP suppresses breast cancer growth in the bone [16,17].

The relationships between OS, reactive oxygen species (ROS), and the tumor are one of the primary focuses in cancer research. ROS affects genomic stability and alters gene expression during tumorigenesis and is widely accepted as one of the major causes of cancer [18]. Furthermore, ROS could change the microenvironment during tumor initiation and progression. However, the mechanistic role of OS in tumor growth and metastasis remains largely unknown. Moreover, there is scarce research focusing on the OS microenvironment and cancer bone metastasis.

This study reports a novel role of osteocytes and Cx43 hemichannels in breast cancer growth in bone under an OS environment. We show that elevated OS inhibited tumor cell growth, and Cx43 hemichannels likely mediated this effect in osteocytes. Impairment of Cx43 hemichannels, either by a hemichannel blocking antibody in vitro under OS or in Cx43 transgenic mice under estrogen deficiency elevated OS level, led to the inhibition of tumor growth. Treatment with an antioxidant reversed the effect, increasing tumor growth in mice with impaired hemichannels. This study unravels an underlying mechanism regarding the role of osteocytic Cx43 hemichannels in modulating an oxidative bone microenvironment and the growth response of breast cancer cells to local OS.

## 2. Results

### 2.1. OS Inhibits Migration of Tumor Cells

**Figure 1:**
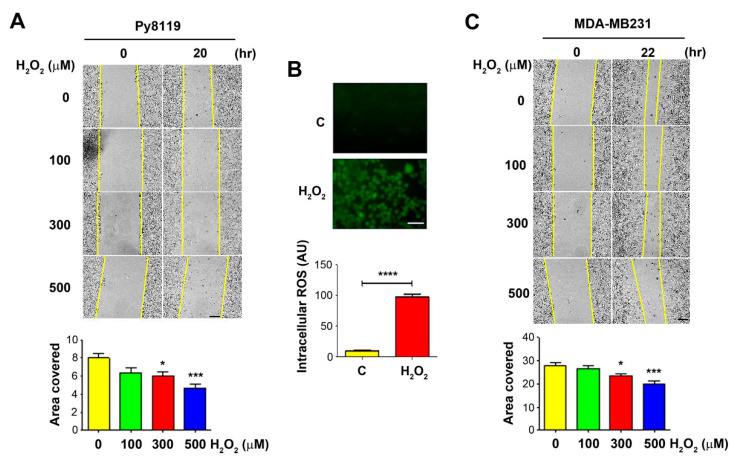
Decrease of breast cancer cell migration by OS. A breast cancer cell, Py8119, (**A**) or MDA-MB-231 (**C**). For (**A**,**C**), bar = 200 μm, wound healing assay was performed (upper panels), and cells were treated with PBS control or 100, 300, and 500 μM H_2_O_2_ for 20–22 h. The extent of the migration was quantified by the covered migration areas using NIH ImageJ software (lower panels) and normalized based on the initial images to obtain the covered area. Py8119 cells were treated with 300 μM H_2_O_2_ for 4 h and followed by incubating with florescence carboxy-H_2_DCFDA for the detection of intracellular ROS levels, bar= 50 μm (**B** upper panels). At least three microphotographic fields were captured under a 20× microscope with an FITC fluorescence filter. The average pixel density of 30 random cells was measured using NIH ImageJ software and quantified (**B,** lower panels). The data are presented as mean ± SEM, *n* = 3. Compared to PBS control (C or 0 µM H_2_O_2_), * *p* < 0.05; *** *p* < 0.001; **** *p* < 0.0001 (*t*-test and one-way ANOVA).

To determine the effect of OS on tumor cell migration, we performed the wound-healing assay with breast cancer cells in the presence of H_2_O_2_ at 100, 300, and 500 μM. We used murine triple-negative breast carcinoma Py8119 cells. The wounded cell areas were measured before the treatment and at 20–22 h post-treatment. The results showed that, compared to the control PBS-treated group, cells treated with H_2_O_2_ migrated slower, showing fewer coverage areas of Py8119 cells (Figure 1A, upper panel). Quantification of the wound areas showed a significant decrease in cell migration for Py8119 cells at 300 and 500 µM (Figure 1A, lower panel). Treatment with 300 µM H_2_O_2_ for 4 h significantly increased ROS levels in Py8119 cells, as determined by a fluorescence-based carboxy-H_2_DCFDA assay (Figure 1B). A similar dose-dependent reduction of cell migration was also observed in human breast cancer MDA-MB-231 cells, with the greatest reduction at 500 µM H_2_O_2_ (Figure 1C).

### 2.2. Osteocytes Support Breast Cancer Cell Growth under High OS Levels

**Figure 2:**
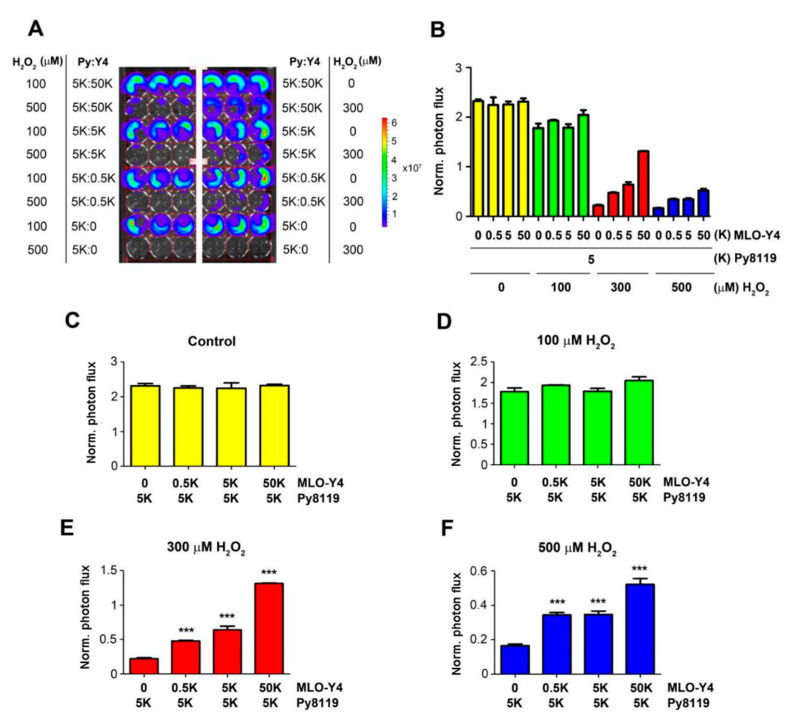
Osteocytes protect breast cancer cell growth in a dose-dependent manner under high OS. Different amounts of MLO-Y4 cells (50K, 5K, and 0.5K) were co-cultured with 5K luciferase-labeled Py8119 cells in 48-well culture plates under PBS (vehicle control) or treated with 100, 300, and 500 μM H_2_O_2_ for 24 h. Cancer cell growth was determined by bioluminescence signals (**A**). The corresponding photon flux of bioluminescence signals from Py8119 cells was quantified (**B**). The photon flux of Py8119 cells in co-culture was quantified under the treatment of control (**C**) or 100 μM (**D**), 300 μM (**E**), or 500 μM (**F**) H_2_O_2_. The photon flux level was normalized with respect to that of the first day. *** *p* < 0.001. The data are presented as mean ± SEM, *n* = 3 (one-way ANOVA).

To determine the impact of the bone microenvironment on cancer cells, we focused on osteocytes, the most abundant cell type in bone tissue. We co-cultured Luc-GFP expressing Py8119 cells with osteocyte MLO-Y4 cells with a fixed number of cancer cells (five thousand (5K)) and different numbers of osteocytes (0.5K, 5k, and 50K) (Figure 2). The co-cultured cells were treated with H_2_O_2_ at concentrations of 100, 300, and 500 µM for 24 h. Py8119 cell growth was monitored by photon flux of bioluminescence signals (Figure 2A) and quantified (Figure 2B). The results showed that co-culturing with osteocytes did not increase Py8119 cell growth even with 10 times more osteocytes (50K) (Figure 2C). Similarly, osteocytes had minimal impact on cancer cell growth at low concentrations of H_2_O_2_ at 100 µM (Figure 2D). However, with H_2_O_2_ at 300 and 500 µM, Py8119 cell growth was significantly increased in the presence of osteocytes (Figure 2E,F). Moreover, the cancer cell growth was positively correlated with the numbers of co-cultured osteocytes under 300 or 500 µM H_2_O_2_, conditions with the greatest increase of cancer cell growth in the presence of 10 times more MLO-Y4 cells (50K).

**Figure 3:**
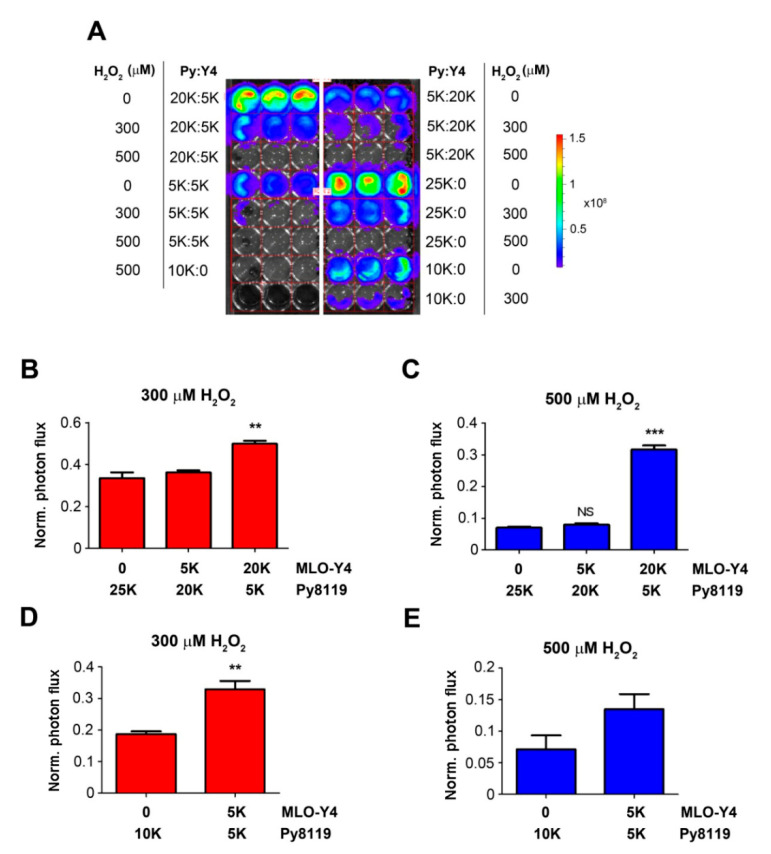
High ratio of osteocytes to breast cancer cells enhances the protection of cancer cell growth under high OS. A total of 25K or 10K cells with various ratios of MLO-Y4 to Py8119 cells were co-cultured in 48-well culture plates under PBS vehicle (control) or treated with 300 or 500 µM H_2_O_2_ for 24 h (**A**). Cancer cell growth was determined by photon flux of bioluminescence signals. Photon flux in the corresponding co-culture was quantified under 300 μM (**B**,**D**) and 500 μM H_2_O_2_ (**C**,**E**) conditions. The photon flux level was normalized with respect to that of the first day and also based on the control group under the same condition. The data are presented as mean ± SEM, *n* = 3. ** *p* < 0.01 and *** *p*< 0.001 (*t*-test and one-way ANOVA).

To exclude the possibility that the effect of osteocytes was caused by the difference in total numbers of co-cultured cells, we kept the total numbers of cells the same (25K or 10K), with various ratios of Py8119 and MLO-Y4 cells (Figure 3). Consistent with the results shown in Figure 2, co-culturing with more MLO-Y4 cells (20K, four times more than Py8119 cells) at 300 and 500 µM H_2_O_2_ significantly increased Py8119 cell growth (Figure 3B,C). The increase of cancer cell growth was also observed with the same total numbers of osteocytes (10K) and cancer cells at 300 and 500 µM H_2_O_2_ (Figure 3D,E). Together, these studies showed that breast cancer cell growth was inhibited under high levels of OS, but this inhibitory effect was compromised in the presence of osteocytes. The data suggest a supporting role of osteocytes in breast cancer growth in response to high OS.

### 2.3. Cx43 Hemichannels in Osteocytes Protect Cancer Cell Growth under High OS

**Figure 4:**
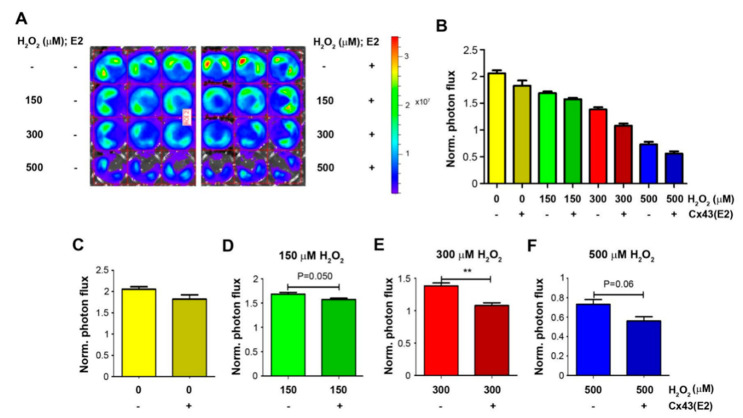
Cx43 hemichannels in osteocytes protect breast cancer cell growth under high OS. In total, 20K MLO-Y4 cells and 5K Py8119 cells were co-cultured and treated with various concentrations of H_2_O_2_ (150, 300, and 500 µM) in the absence or presence of Cx43E2 antibody for 24 h, and tumor cell growth was determined by photon flux of bioluminescence (**A**) and quantified (**B**). In addition, these results were compared under certain concentrations of H_2_O_2_ in the absence or presence of Cx43E2 (**C**–**F**). The photon flux level was normalized with respect to that of the first day. The data are presented as mean ± SEM, *n* = 3. ** *p* < 0.01 (*t*-test).

Cx43 hemichannels opened by OS are shown to protect from oxidation-induced osteocyte cell death [15]. Thus far, we have demonstrated that osteocytes protect cancer cell growth under high OS, and here, we further assessed the involvement of osteocytic Cx43 hemichannels in the role of osteocytes under high OS. Luc-labeled Py8119 cells (5K) were co-cultured with MLO-Y4 cells (20K) and treated with 150, 300, and 500 μM H_2_O_2_ and PBS control in the presence of Cx43 hemichannel blocking antibody, Cx43E2. This antibody specifically inhibits Cx43 hemichannels in osteocytes [19] but has minimal effect in Py8110 cells since no Cx43 protein was detected in Py8119 cells (Figure S1). Py8119 cell growth was similarly determined by bioluminescence (Figure 4A), and photon flux signals were quantified (Figure 4B). The treatment with H_2_O_2_ at 150, 300, and 500 μM inhibited Py8119 cancer cell growth when co-culturing with osteocyte MLO-Y4 cells. The inhibition of Cx43 hemichannels by Cx43E2 had minimal effect on cancer cell growth in co-cultured cells (Figure 4C). However, Cx43E2 decreased the protective effect of osteocytes on Py8119 cell growth in co-cultured cells (Figure 4D–F) and reached a significant level with 300 μM H_2_O_2_ (Figure 4E). These results showed that the inhibition of Cx43 hemichannels by Cx43E2 antibody mitigated the protective function of osteocytes on breast cancer cell growth under high OS, suggesting a role of osteocytic Cx43 hemichannels in breast cancer cell growth in response to OS.

### 2.4. The Inhibitory Effect of Cx43 Hemichannels on Breast Cancer Growth in Bone Is Mitigated in Ovariectomized Mice

**Figure 5:**
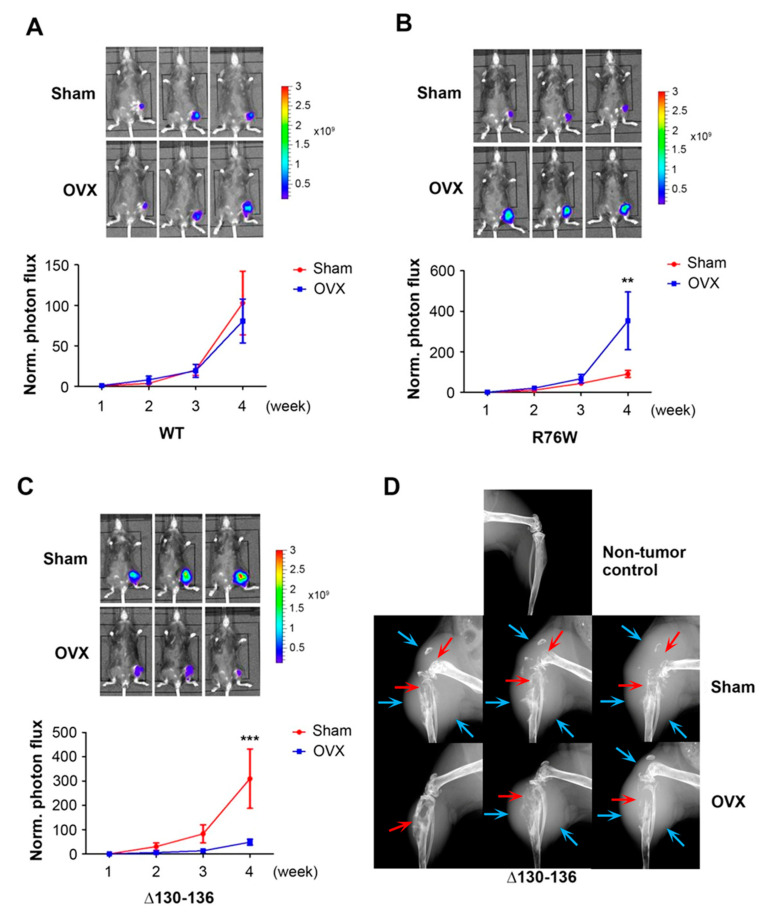
Cx43 hemichannels in osteocytes are involved in intratibial breast cancer growth in OVX mice. Py8119 cells were implanted in the tibia of sham and OVX WT (**A**) and two Cx43 transgenic mice, R76W (**B**) and Δ130-136 (**C**). Tumor growth was monitored weekly, and bioluminescence images were taken 4 weeks after tumor implantation (upper panels). The bioluminescence signals were quantified (lower panel). (**D**) X-ray scan of the legs of Δ130-136 mice 4 weeks after tumor implantation; blue arrows show the border of the tumor and red arrows are pointed at the site of bone destruction. WT sham, WT OVX, R76W sham, R76W OVX, Δ130-136 sham, and Δ130-136 OVX mice, *n* = 6, 8, 12, 13, 7, and 8, respectively. The data are presented as mean ± SEM. ** *p* < 0.01 and *** *p* < 0.001 (two-way RM ANOVA).

To investigate the role of OS and Cx43 hemichannels in breast cancer bone metastasis in vivo, we implanted murine Py8119 breast cancer cells into tibia bone marrow through intratibial injection. We used two transgenic mouse models overexpressing in osteocytes Cx43 dominant-negative mutants: R76W, which dominant-negatively inhibits only gap junctions, and Δ130-136, which dominant-negatively inhibits both gap junctions and hemichannels [13]. Tumor growth was monitored every week with bioluminescence imaging. Consistent with our previous report, Δ130-136 transgenic mice exhibited more tumor growth compared to WT and R76W mice, and there was no difference between WT and R76W mice [17] (Figure 5). OVX mice mimic the post-menopausal process in women. We have previously shown that OVX significantly elevates OS level in bone tissue of Δ130-136 but not in WT and R76W mice [14]. Interestingly, although OVX did not increase breast cancer growth in the bone in WT (Figure 5A), a significant increase was observed in R76W (Figure 5B) at the fourth week after intratibial tumor implantation. The increase of breast cancer growth in OVX mice could be caused by the decrease of Cx43 expression and hemichannel activities, as we have shown previously [14]. In contrast to WT and R76W mice, breast cancer growth in the bone of Δ130-136 mice after OVX was significantly reduced. The tumor growth curve showed much slower growth in the OVX group than in the sham group in Δ130-136 mice, which reached a significant level at week four (Figure 5C). Meanwhile, X-ray scanning showed that the sham group of Δ130-136 mice had a larger-sized tumor and severe bone destruction 4 weeks after tumor implantation, which is consistent with bioluminescence data (Figure 5D). The data suggest that elevated OS in the bone microenvironment caused by OVX in Δ130-136 mice may result in a suppression of breast cancer growth.

### 2.5. Antioxidant Treatment Increases Tumor Growth in Bone of Δ130-136 Mice

**Figure 6:**
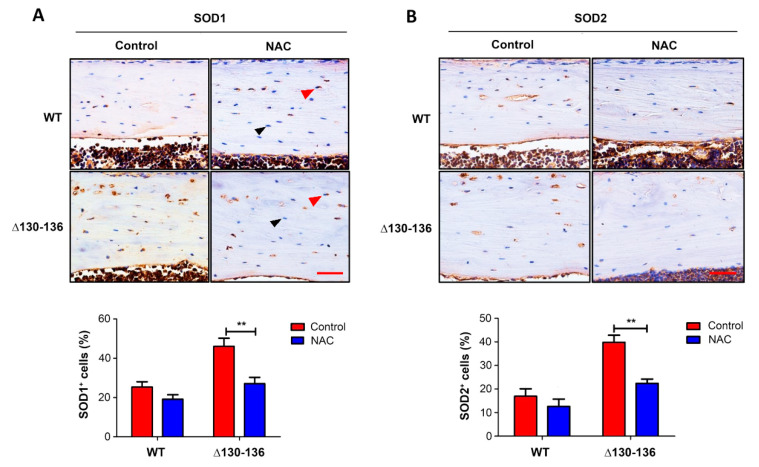
NAC treatment reduces OS in Δ130-136 mice. Paraffin sections of tibia cortical bone were immunolabeled with SOD1 antibody. Red arrows indicate SOD1 osteocytes with positive signals, while black arrows indicate negative signals (upper panel of (**A**)). The percentage of SOD1 positive cells was quantified (lower panel of (**A**)). Paraffin sections of tibia cortical bone were also immunolabeled with SOD2 antibody (upper panel of (**B**)). The percentage of SOD2 positive cells was quantified (lower panel of (**B**)). Scale bar = 50 µm. Data shown as mean ± SEM. ** *p* < 0.05. *n* = 3–5 in each group (two-way ANOVA).

To further manifest the impact of an elevated OS microenvironment and osteocytic Cx43 hemichannels on breast cancer growth in bone, we used antioxidant NAC by intraperitoneal injection twice a week and daily feeding with drinking water. OS level in tibial bone was determined by two OS markers, superoxide dismutase 1 (SOD1 or CuZn-SOD) and superoxide dismutase 2 (SOD2 or Mn-SOD) (Figure 6). Immunohistochemical staining for both SOD1 (Figure 6A) and SOD2 (Figure 6B) showed that the positively stained osteocytes in the Δ130-136 NAC-treated group were significantly fewer than those in the control group; however, no obvious difference was noted between NAC treatment and control in WT mice.

**Figure 7:**
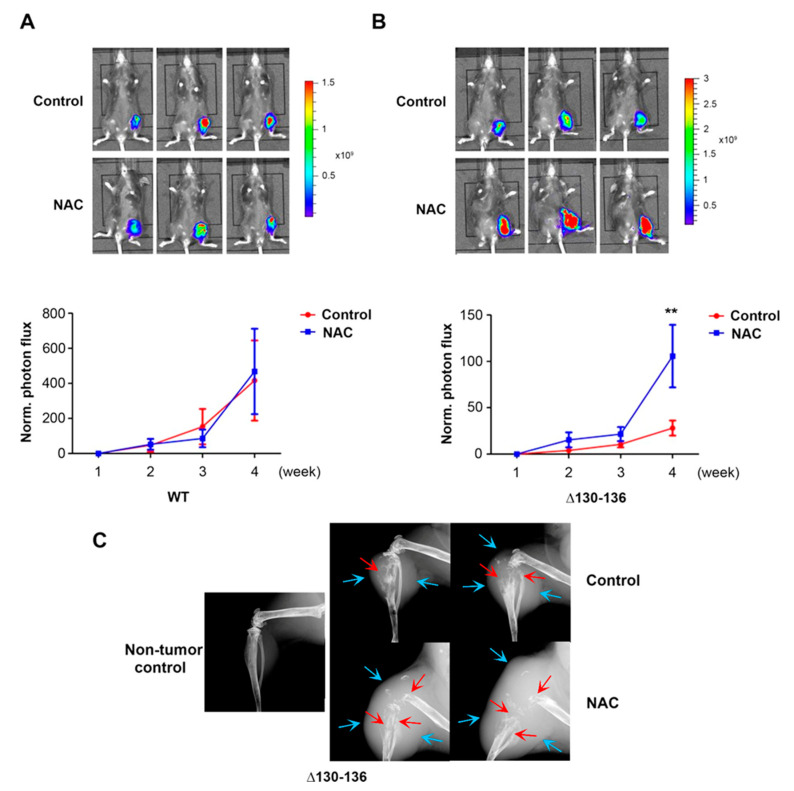
Cx43 hemichannels play a protective role in breast cancer growth in vivo in response to OS. Py8119 cells were implanted in the tibia of 6–8-week-old WT and Δ130-136 mice, and NAC treatment was initiated two days before intratibial injection of Py8119 cells. The mice were fed with an antioxidant, NAC in the drinking water daily and IP injected with NAC twice a week. Tumor growth was monitored weekly and quantified by bioluminescence signals. Tumor growth rate was determined by normalizing photon flux to that of 24 h post-intratibial injection. Bioluminescence images were taken at 4 weeks post-intratibial injection of control and NAC-treated WT mice (upper panel, **A**) and Δ130-136 mice (upper panel, **B**) and normalized tumor growth rate (lower panels, **A** and **B**) were shown. (**C**) X-ray images of the legs of control and NAC-treated Δ130-136 mice at 4 weeks post-intratibial injection. WT control, WT NAC-treated, Δ130-136 control, and Δ130-136 NAC-treated mice, *n* = 8, 7, 5, and 4, respectively. The data are presented as mean ± SEM. ** *p* < 0.01 (two-way RM ANOVA).

We did intratibial tumor implantation in WT and Δ130-136 mice and treated these mice with the antioxidant NAC by intraperitoneal injection twice a week and by daily feeding with drinking water. The tumor burden was quantified by photon flux signals to determine tumor growth in WT (Figure 7A) and Δ130-136 (Figure 7B). Compared to the nontreated control group, a significant increase in the NAC-treated group after 4 weeks was shown in Δ130-136 mice (Figure 7B), while such an increasing trend was not observed in NAC-treated WT mice (Figure 7A). Four weeks after tumor implantation, X-ray imaging also showed increased tumor sizes in the tibia of Δ130-136 mice (Figure 7C). These results were correlated with the OS levels in WT and Δ130-136 and supported the notion that increased OS hindered breast cancer colonized in bone, and Cx43 hemichannels activated by high OS provided a potential protective role on breast cancer colonization in the bone.

## 3. Discussion

The mechanistic role of the oxidative microenvironment over breast cancer cells in the bone remained largely elusive and somewhat controversial [20,21]. Previous studies show different effects at different pathological stages in the primary breast and the distal metastasized organs [22]. Early studies have shown that ROS is highly correlated with tumorigenesis by affecting the stabilization of genes [18]. However, more recent studies found that the accumulation of oxidative stress could be harmful and even deadly for cancer cells [23]. Most interestingly, the oxidative level and the ability of cancer cells to resist OS are different at different stages of cancer development [22]. Osteocytes, the most abundant cells in the bone tissue, are a key player in bone homeostasis, regulating bone remodeling and quality [13]. The survival of osteocytes relies on the normal function of Cx43 hemichannels [13,15]. Estrogen is known to reduce oxidative stress in the bone. Under estrogen deficiency, often seen in post-menopausal women or patients with ovariectomy, the function of the hemichannels is reduced, leading to a bone microenvironment with elevated oxidative stress [14]. In this paper, for the first time, we provide evidence in vivo and in vitro that functional Cx43 hemichannels protect the bone microenvironment against oxidative stress and that protection preserves the growth of breast cancer cells.

We show in this study that elevated OS due to H_2_O_2_ inhibits breast cancer cell migration and growth, and co-culturing with bone osteocytes protects breast cancer cells from OS. During our wound-healing assay, proliferation or viability of cells was limited because cells were 95% confluent, and the difference showed in the result was primarily caused by the migration and cell death. The impairment of osteocytic Cx43 hemichannels in transgenic mouse models in vivo elevates OS in the bone microenvironment and suppresses tumor burden in OVX mice, while antioxidant NAC reduces OS and enhances tumor growth. This study unveils a new underlying mechanism in which Cx43 hemichannels in osteocytes play an important role in regulating bone redox hemostasis and highlights their impact on breast cancer bone growth.

A major underlying question is how osteocytes modulate OS in the bone microenvironment and, concurrently, tumor growth. One of the potential candidates that could mediate this is Cx43 hemichannels. Cx43 hemichannels are richly present on the surface of osteocytes [12,24]. These channels are usually closed under normal physiological conditions; however, certain stress conditions induce the opening of hemichannels [25,26,27]. We have previously shown that the opening of Cx43 hemichannels is a self-protective mechanism that promotes the survival of osteocytes [15]. By using a specific Cx43 hemichannel blocking antibody [19], we show that inhibition of the hemichannels reduces the protective effect of osteocytes on breast cancer cells under OS. Cx43 hemichannels in osteocytes release ATP that activates purinergic signaling and inhibits breast cancer cell growth and migration, while adenosine, a product of ATP, promotes cancer growth [16,17]. Under oxidative stress, cells will generate less ATP associated with more adenosine [28]. Thus, osteocytes are expected to release less ATP and more adenosine. The relatively higher level of adenosine is likely to promote cancer growth. However, the inhibition of tumor growth by OVX and the promotion of tumor growth induced by NAC in Δ130-136, but not in WT and R76W mice, strongly suggest that the high oxidative stress level in the bone of Δ130-136 inhibits tumor growth. The lack of NAC’s effect on WT mice suggests that the reducing state of the bone microenvironment is sufficient to sustain a normal microenvironment for breast cancer growth, and osteocytic Cx43 hemichannels play an important role in maintaining redox homeostasis in the bone microenvironment. This evidence points to the roles of Cx43 hemichannels in regulating an oxidative microenvironment and breast cancer cell growth.

OVX surgery in mice is a commonly adopted animal model that mimics the estrogen deficiency in post-menopausal women [29,30], which is associated with an imbalance in intraosseous redox [31,32]. We reported earlier that, after estrogen withdrawal, the expression levels of Cx43 and Cx43 hemichannel activity in osteocyte MLO-Y4 cells are decreased [14]. In this study, we used an OVX mouse model to determine the growth of breast cancer cells under an OS microenvironment in the bone. We previously found that oxidative stress levels of WT, R76W, and Δ130-136 mice have no significant difference. However, after OVX, the oxidative stress level increases significantly in the bones of Δ130-136 mice but not in the bones of WT and R76W mice [14]. The evidence strongly suggests that the increased oxidative stress level found in the bones of Δ130-136 mice is likely caused by the impairment of the Cx43 hemichannel function, because no increase in oxidative stress was found in WT and R76W mice. The inhibition of the intratibial Py8119 tumor growth observed in OVX Δ130-136 could likely be attributed to the unfavorable microenvironment induced by the damage from accumulative oxidative stress. In agreement, the increased tumor growth observed in OVX treated WT and R76W mice could be caused by increased bone resorption because of OVX-induced estrogen deficiency [33,34] but is unlikely to be caused by elevated oxidative stress, because we did not detect increased oxidative stress levels in the tibia of these animal models. Consistent with our in vitro results, a high oxidative stress level has an inhibitory effect on tumor growth.

We further validated the impact of oxidative stress on Δ130-136 mice by using a reductant NAC, a commonly used antioxidant [35,36,37]. Although NAC has a minimal effect on tumor growth for WT, it greatly promotes tumor growth in Δ130-136 mice. These data from both in vitro and in vivo assays help elucidate the roles of osteocytic Cx43 hemichannels in the redox regulation of the bone microenvironment and consequently in breast cancer bone metastases. Published studies show contradictory evidence regarding the efficacies of antioxidant adjuvant drugs in cancer prevention and cancer treatment in various cancer types [22,38,39,40,41,42,43,44,45,46]. It is likely that OS may play different roles depending upon the microenvironment of the host tissue [18,23]. We evaluated OS levels in NAC-treated bone samples with two OS markers, SOD1 and SOD2, a family of enzymes that catalyze the dismutation of superoxide anions [47]. We showed that NAC only decreased SOD1 and SOD2 expression in Cx43-hemichannel-impaired Δ130-136 mice but not in WT mice. Moreover, our previous data showed that OVX surgery only increases OS level in Δ130-136 mice but not in WT and R76W mice [14]. Therefore, the suppression and promotion of tumor growth in OVX and NAC-treated mice, respectively, were consistent with changes of the OS levels in these models due to the impaired Cx43 hemichannels in osteocytes.

**Figure 8:**
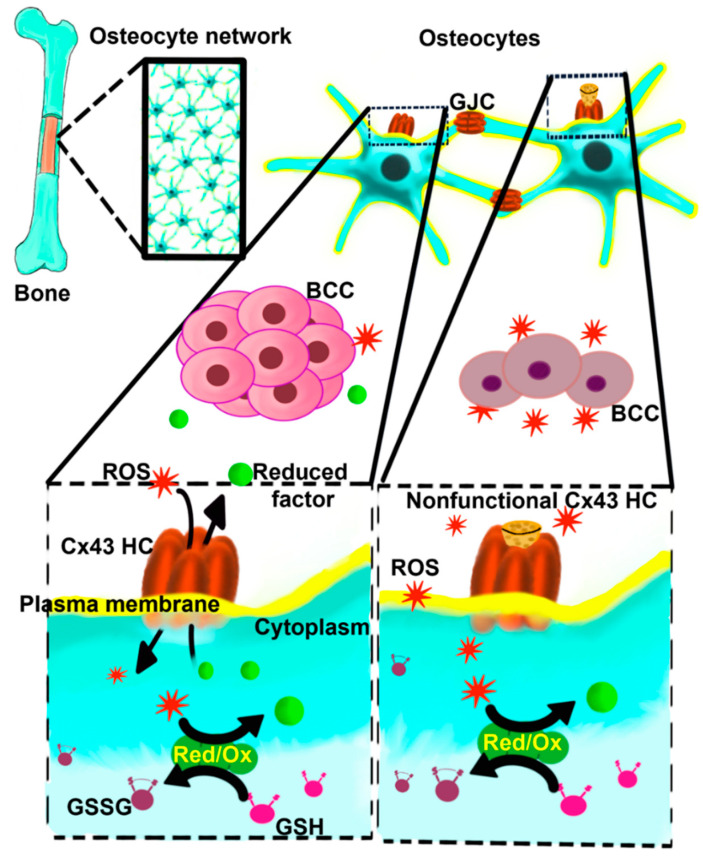
Schematic model of the role of Cx43 HCs in regulating the oxidative bone microenvironment and breast cancer growth. Osteocytes immersed in the bone mineral matrix communicate through gap junction channels (GJCs), forming an extensive osteocyte network. Osteocytes express Cx43 HCs on the cell surface. The opening of Cx43 HCs allows the exchange of oxidant/reductant molecules from the bone environment, and these molecules are metabolized and neutralized by the Red/Ox enzyme machinery present in the osteocytes, using endogenous resources such as glutathione (GSH) and forming oxidated products as oxidized GSH (GSSG). Reduced factors and other metabolites produced could be released from the osteocyte forming a propitious environment permissible for metastatic breast cancer cell (BCC) growth. The lack of Cx43 HC function (nonfunctional Cx43 HC) allows the accumulation of ROS in the extracellular bone environment, inhibiting the BCC growth and reducing the survival of bone cells and the bone quality.

This study cannot completely rule out the possible involvements of Cx43 other than through hemichannels, such as the channel-independent function of Cx43 or the role of Cx43 in mitochondria. However, the possible involvement of gap junctions is likely minimal. First, Cx43E2 antibody specifically inhibits hemichannels but not gap junction function, as reported by multiple studies [12,15,19,48,49]. Our in vivo transgenic models further validate in vitro cell studies. R76W mice that had impaired gap junctions in osteocytes with enhanced hemichannels, showed opposite results compared to Δ130-136 mice with impaired gap junction channels and hemichannels. We observed the effects on cancer both in vitro and in vivo with the specific inhibition of hemichannels, which consistently suggests the roles of Cx43 hemichannels. Moreover, previous studies using both Cx43 siRNA and Cx43E2 antibody showed consistent results of Cx43 and Cx43 hemichannels in protecting osteocytes against oxidative-stress-induced cell death [15]. Taken together, as illustrated in Figure 8, this study provides a mechanistic implication concerning the impact of osteocytes and Cx43 hemichannels on the bone oxidative microenvironment and breast cancer progression and metastasis. It may further offer an insightful indication regarding the cautious use of antioxidants in cancer therapy.

## 4. Materials and Methods

### 4.1. Cell Culture

Murine MLO-Y4 and Py8119 cells were obtained from Lynda Bonewald at Indiana University and Lesley Ellies at University of California at San Diego, respectively. Both cell lines were recently authenticated with expression of marker proteins and tested for mycoplasma contamination. MDA-MB-231 cells were obtained from ATCC. MLO-Y4 cells were cultured on collagen-coated (0.15 mg/mL, rat tail collagen type I, BD Biosciences, Franklin Lakes, NJ, USA, 354236) surfaces and were grown in α-minimum essential (α-MEM) medium supplemented with 2.5% fetal bovine serum and 2.5% bovine calf serum. Py8119 cells were grown in F12K medium supplemented with 5% fetal bovine serum and 1 μL/mL MITO (Corning™ MITO+ Serum Extender, Fisher Scientific, Waltham, MA, USA, CB-50006). Human breast carcinoma MDA-MB-231 cells were grown in McCoy’s 5A medium supplemented with 10% fetal bovine serum. All cells were incubated in a 5% CO_2_ incubator at 37 °C.

### 4.2. Measurement of Intracellular ROS 

Intracellular ROS levels in cells were determined using the fluorescence-based probe carboxy-H_2_DCFDA (Invitrogen, Carlsbad, CA, USA) in live cells. Briefly, cells were rinsed with HBSS and incubated with 10 μM carboxy-H_2_DCFDA for 30 min at 37 °C. The cells were rinsed and maintained in HBSS followed by fluorescence analysis. At least three fields of microscopic view (Olympus, Tokyo, Japan) were captured, and the average pixel density of 30 random cells from each field was measured using NIH ImageJ 64-bit Java 1.8.0_172 software.

### 4.3. Wound-Healing Assay 

Py8119 or MDA-MB-231 cells were cultured in 100 mm culture plates for 5 days (~95% confluence) with a change of medium every two days. The last 24 h medium was collected as conditioned medium, filtered by a 0.22 μm filter, and then stored at 4 °C (less than 24 h). These conditioned media were collected from the same cell line used for the wound-healing migration assay. Fresh cells were seeded on a 12-well sterile cell culture plate and incubated for 24 h at 37 °C. Then, a scratch wound across the middle of each well was made using a sterile 200 μL pipette tip. Immediately after wounding, the cells were rinsed, the initial images were taken, and the location of the wound was marked to take images of the same place. Conditioned medium was then added along with PBS or various concentrations of H_2_O_2_. Images of the marked area were then taken 20–22 h after the treatment. Cell migration was quantitatively measured and presented as the percentage of the migrated (covered) area by first subtracting the final gap areas from the initial ones, and the sum was divided by the initial gap areas using NIH ImageJ software.

### 4.4. Cancer Cell Growth under Co-Culturing with Osteocytes 

MLO-Y4 cells and Py8119 cells expressing Luc-GFP were cultured separately with their corresponding culture media on collagen-coated (0.15 mg/mL, rat-tail collagen type I, BD Biosciences, 354236) surfaces. MLO-Y4 media was collected after 24 h of culture, filtered using a 0.22 μm filter, and stored at −20 °C (less than a week). Various numbers or ratios of MLO-Y4 cells and Py8119 cells were transferred to a 48-well sterile cell culture plate coated with collagen and grown in α -MEM media for 24 h. The initial bioluminescence images were taken with luciferase and IVIS Lumina system (Andor Technology, Belfast, Northern Ireland, Model NO: DS934N-BV-286). The media were then removed and replaced with MLO-Y4-conditioned media and various concentrations of H_2_O_2_ and 3 μg/mL Cx43E2 antibody or PBS for another 24 h. The images were then captured similarly to the initial images. Cell growth was quantitatively measured by photon flux and normalized to the value of the initial images. The result was also normalized to the prior corresponding baseline, when appropriate. If the number of Py8119 cells differed between groups, data were renormalized by using the first-time normalized result divided by the averaged photon flux of the control group, which had the same number of Py8119 cells under the same condition, to make the results comparable.

### 4.5. Animal Models 

We established two transgenic mouse models overexpressing dominant-negative Cx43 mutants, R76W and Δ130-136 [13]. We used 6- to 8-week-old female C57BL/6 J WT and transgenic mice. All transgenic mice used in this study were homozygous and bred separately. Each mouse in a cohort was tagged by unique ear cutting. Mice were randomly divided into different groups (4–13 animals/group) and experiments were conducted blindly. We excluded animals in the analysis due to tumor implantation errors and unexpected animal death. All animal protocols were reviewed and approved by the University of Texas Health Science Center at San Antonio (UTHSCSA) Institutional Animal Care and Use Committee (IACUC).

### 4.6. Ovariectomy (OVX) Procedure 

Ovariectomy was performed largely based on a previously published protocol [50]. Briefly, 6-week-old mice were randomly separated into OVX and sham groups. Before the operation, mice were anesthetized by intraperitoneal injection of 100 mg/kg of ketamine (Butler Schein, Dublin, OH, USA) and 16 mg/kg of xylazine (Butler Schein). A small incision over the ovaries was made, and the ovaries with fat pads were held by a hemostat and cut by a scalpel after a knot was made under the fat pad. The uterine horn was double-checked to make sure that no damage was done, and the ovaries were completely removed. The mice had 4 weeks of recovery from OVX surgery and were kept housed in a temperature-controlled room with a light/dark cycle of 12 h. All mice were given food and water ad libitum.

### 4.7. Antioxidant Treatment for Mice 

All 6–8-week-old mice were randomly divided into control and treatment groups. Before intratibial injection of tumor cells, mice were administrated 100 mg/kg N-acetylcysteine (NAC) in PBS each day by intraperitoneal (IP) injection for two days. After tumor implantation, NAC was injected twice per week. Additionally, mice were provided with 1 g/L NAC in drinking water, and the water was changed every week. For the control vehicle group, the same volume of PBS was injected, and normal drinking water was provided. The solutions used for injection were filtered and sterile, and the pH of the PBS solution was 7.3–7.4. NAC water was prepared freshly before the treatment.

### 4.8. Tumor Cell Intratibial Injection, Bioluminescence, and X-ray Imaging 

Py8119 cells expressing Luc-GFP were used for tumor implantation. Cultured Py8119 cells were re-suspended with PBS at 500,000 cells/mL. Mice were anesthetized using a gas anesthesia system (XGI-8 Gas Anesthesia System, Xenogen, Alameda, CA, USA) with isoflurane. A 0.5 cm incision was made to expose the knee of the left side after the removal of leg hairs. Twenty microliters of the solution containing Py8119 cells was injected into the left tibia of each mouse by a microinjector (GASTIGHT#1705, Hamilton, Bonaduz, Switzerland), and the incision was sutured. Tumor growth was monitored with a bioluminescence imaging system (Xenogen IVIS spectrum imaging system), which was initiated a week after intratibial implantation of PY8119 cells, and the scanning was conducted weekly. Tumor growth was analyzed using a Living Image software (Xenogen, Caliper Life Sciences, Hopkinson, MA, USA) by measuring the intensity of photon flux (photons/s/cm^2^/steradian). Tumor burden was expressed as photon flux in a standardized region of interest surrounding the major bioluminescence signal and normalized to the value at day 7. X-ray imaging was performed with a Faxitron X-ray model MX20/DX50 (Wheeling, IL, USA), measurement was conducted one or two days after the 4th bioluminescence scan.

### 4.9. Preparation of Bone Tissue Sections and Immunohistochemistry

Immunohistochemistry staining was performed using anti-SOD1 and SOD2 antibodies to detect OS levels. Briefly, mice were sacrificed 5 weeks after tumor implantation, and right tibia samples were fixed by 4% PFA for two days and then decalcified thoroughly by daily change of buffer, 10% EDTA w/v in water (pH = 7.5), for three weeks. Tibia paraffin sections were antigen unmasked by using sodium citrate buffer (pH = 6.0) and treated with normal serum to block nonspecific binding. Tibia bone tissue sections were labeled with anti-SOD1 polyclonal antibody (1:100 dilution, sc-11407, Santa Cruz, Dallas, TX, USA) and anti-SOD2 polyclonal antibody (1:150 dilution, sc-30080, Santa Cruz) at 4 °C overnight. The sections were then followed by biotin-labeled secondary antibody and signal amplification, DAB kit (Vector, Burlingame, CA, USA) according to manufacturer’s protocols. Hematoxylin was applied for nuclear counterstain. The stained tissue sections were photographed, and the percentage of SOD1- and SOD2-positive cells was quantified using NIH ImageJ software.

### 4.10. Statistical Analysis 

All figures and statistical analyses were performed using GraphPad Prism 5 statistical software (GraphPad Software Inc., San Diego, CA, USA). For animal studies, 4–13 animals per group were chosen, which provided adequate power to detect significant changes. All data are presented using mean ± standard errors ($\bar{X}$ ± SEM). Two groups in cell experiments were analyzed with *t*-test, and multiple groups were analyzed with one-way analysis of variance (one-way analysis) with Dunnett’s multiple comparison test. The weekly repeated measurements of animal tumor growth results were statistically analyzed by two-way repeated measurement analysis of variance (two-way RM analysis). The variance was similar between the groups that were being statistically compared. Asterisks indicate significant differences compared to controls (* *p* < 0.05; ** *p* < 0.01; *** *p* < 0.001; **** *p* < 0.0001).

## 5. Conclusions

Our research showed that oxidative stress inhibits breast cancer cell growth both in vitro and in vivo, and Cx43 hemichannels play a critical role in breast cancer growth under OS bone microenvironment. Antioxidant promotes cancer growth in osteocytic Cx43 channel-impaired mice.

## Data Availability

The data presented in this study are available in the article and Supplementary Material.

## Supplementary Materials

The following are available online at https://www.mdpi.com/article/10.3390/cancers13246343/s1, Figure S1. Cx43 expression in Py8119 and MLO-Y4 cells.
