# Supplementary material for: Osteocytic Connexin Hemichannels Modulate Oxidative Bone Microenvironment and Breast Cancer Growth
Source: Cancers (Basel). 2021 Dec 17;13(24):6343. doi: 10.3390/cancers13246343 (PMC8699531; doi:10.3390/cancers13246343)

Supplemental Figure Legends

Figure S1. Cx43 expression in Py8119 and MLO-Y4 cells. Crude membrane extracts of Py8119 and MLO-Y4 cells were immunoblotted using anti-Cx43 antibody.

Figure S1

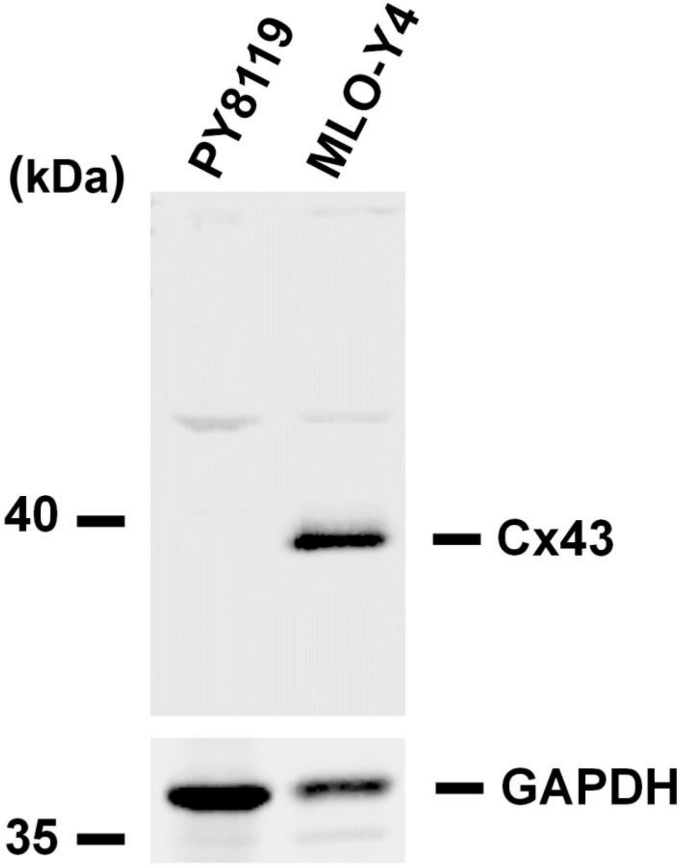

Supplement: Supplementary file 1 [file cancers-13-06343-s001.zip › cancers-1482699-supplementary.pdf]
